# Epidemiology of tuberculosis in Minas Gerais, Brazil, between 2013 and 2023 and the impact of the COVID-19 pandemic

**DOI:** 10.3389/fpubh.2025.1642015

**Published:** 2025-08-20

**Authors:** Renan Clímaco do Bem Braga, Igor Rosa Meurer, Maisah Meyhr D’Carmo Sodré, Luciana Debortoli de Carvalho, Lauro Juliano Marin, Marcelo Silva Silvério, Patrícia Guedes Garcia

**Affiliations:** ^1^Department of Pharmaceutical Sciences, Faculty of Pharmacy, Federal University of Juiz de Fora, Juiz de Fora, Brazil; ^2^Teaching and Research Management, University Hospital of the Federal University of Juiz de Fora/Brazilian Company of Hospital Services, Juiz de Fora, Brazil; ^3^Department of Biological Sciences, Santa Cruz State University, Ilhéus, Brazil; ^4^Department of Clinical Medicine, Faculty of Medicine, Federal University of Juiz de Fora, Juiz de Fora, Brazil; ^5^Department of Health, Santa Cruz State University, Ilhéus, Brazil

**Keywords:** tuberculosis, *Mycobacterium tuberculosis*, COVID-19, epidemiology, underreporting, bacterial drug resistance, public health surveillance

## Abstract

**Background:**

Tuberculosis (TB), a disease caused by bacteria of the *Mycobacterium tuberculosis complex* (MTC), is one of the oldest diseases in human history, and despite several global efforts to reduce case numbers, it remains one of the main causes of death worldwide due infectious agents. This study aimed to analyze the epidemiological trends of tuberculosis in Minas Gerais, Brazil, from 2013 to 2023, with emphasis on the impact of the COVID-19 pandemic on case notification.

**Methods:**

Based on epidemiological data obtained from the DATASUS platform, spanning the period from 2013 to 2023, the number of cases, the distribution of confirmed cases by sex, race, education, age group, HIV co-infection and presence of comorbidities such as diabetes, and risk factors like smoking and alcoholism were evaluated. Additionally, the municipalities with the highest number of confirmed cases were identified.

**Results:**

The research revealed a steady annual rise in TB cases, having the highest number of cases in 2023, with 12.55% of all reported cases. Men between 25 and 54 years of age, with a lower educational level, were the most affected by the disease. Regarding race, the majority of the reported cases were attributed to Brown-skinned people. The co-infection rate involving TB and HIV increased proportionally to the reported cases of TB statewide. Regarding comorbidities and risk factors, diabetes, smoking, and alcoholism composed a large part of the tuberculosis caseload, with alcoholism and smoking being especially related to the male population.

**Conclusion:**

The results reinforced the gravity of tuberculosis as a public health challenge, while highlighting the impact of the COVID-19 pandemic on underreporting and the subsequent increase in reported cases of drug resistance involving tuberculosis.

## 1 Introduction

Tuberculosis (TB) is a bacterial infection caused by bacteria of the *Mycobacterium tuberculosis complex* (MTBC), with *Mycobacterium tuberculosis* being the species commonly associated with the disease. TB represents one of the greatest challenges in public health, infecting more than 10 million people every year. According to the World Health Organization (WHO) (1), in 2022, tuberculosis was the second leading cause of death from a single infectious agent worldwide, just behind the *Coronavirus Disease 2019* (COVID-19), and causing almost twice as many deaths as the human immunodeficiency virus (HIV).

According to The End TB Strategy (2), a program proposed by the WHO in 2014 aiming to end the global tuberculosis epidemic by 2035, it is necessary to rely on three main pillars: patient-centered care and prevention; bold policies and support systems; and accelerated research and innovation. Tuberculosis is a disease strongly related to poverty. The WHO (1) lists 30 countries with the highest burden of TB, HIV co-infection and multidrug-resistant TB (MDR-TB), which are projected to represent 90.0% of tuberculosis cases in the world by 2025. Of these, 24 countries can be considered as developing nations, while Brazil, China, Russia, Thailand, Vietnam, and Ukraine are considered emerging economies, or in the process of socioeconomic transition.

In Brazil, the Tuberculosis Epidemiological Bulletin (3) reports that the drop in the number of rapid molecular testing and the case reporting of people with TB since March 2020 are direct consequences of the COVID-19 pandemic. This allowed us to observe in the following years not only the gradual recovery of these notifications but also the rise in TB incidence. According to WHO (4), COVID-19 has already reached the mark of more than 770 million infected people, being the third major viral pandemic after HIV/AIDS and the Spanish Flu. This pandemic imposed the need for social isolation and caused the overload of healthcare systems, resulting not only in the drop in notifications, but also prioritizing eliminating COVID-19 in relation to other diseases that can be treated by public health, such as tuberculosis, with diverted resources and supplies impacting TB essential services (5).

This research is justified by the persistently high rates of *Mycobacterium tuberculosis* infections and the growing prevalence of rifampicin-resistant TB (RR-TB), which led the World Health Organization to classify this microorganism as a priority pathogen in 2024 (6). Given the global tuberculosis problem, especially in Brazil, and the differences in prevalence between regions, this study aimed to describe the epidemiology of tuberculosis in Minas Gerais, Brazil, over a 10-year period, from 2013 to 2023, with an emphasis on temporal trends before and after the onset of the COVID-19 pandemic.

## 2 Methods

A retrospective analysis of data on TB in Brazil was conducted by accessing the public domain database of the Department of Informatics of the Unified Health System (DATASUS) and the Information System of the Unified Health System (SIH/SUS), of the Ministry of Health, through the electronic address,^1^ covering the period from January 2013 to December 2023. The research was carried out with public domain data and did not require ethical approval from the National Research Ethics Committee (CEP/CONEP System) as described in resolution 466/2012.

Epidemiological data on TB cases in Minas Gerais were collected, evaluating the following parameters: total case numbers; municipalities with the highest number of confirmed cases; distribution of confirmed cases by sex, race, educational attainment and age group; HIV co-infection and presence of comorbidities and risk factors such as diabetes, smoking and alcoholism. Cases of tuberculosis confirmed by laboratory diagnosis were included in this study. The data were manually extracted from the system of the Department of Information Technology of the Unified Health System (DATASUS), specifically from Tabnet/SINAN, using the tools available on the public access platform. Regarding missing data, variables with unfilled fields or those reported as “unknown” were identified. This information was maintained in the analysis as a separate category (“unknown”/“not reported”) whenever possible and reported in the results tables. In cases where missing data compromised the statistical analysis, the analysis was based on valid values (analysis per available case).

The research was conducted with public domain data and did not require ethical approval from the CEP-CONEP System, as described in resolution 466/2012. It used exclusively secondary public data made available by DATASUS, which is anonymized and aggregated, ensuring the privacy and confidentiality of individual information. Therefore, there was no access to identifiable or sensitive data, which exempts the need for approval by a Research Ethics Committee.

## 3 Results

**Figure 1:**
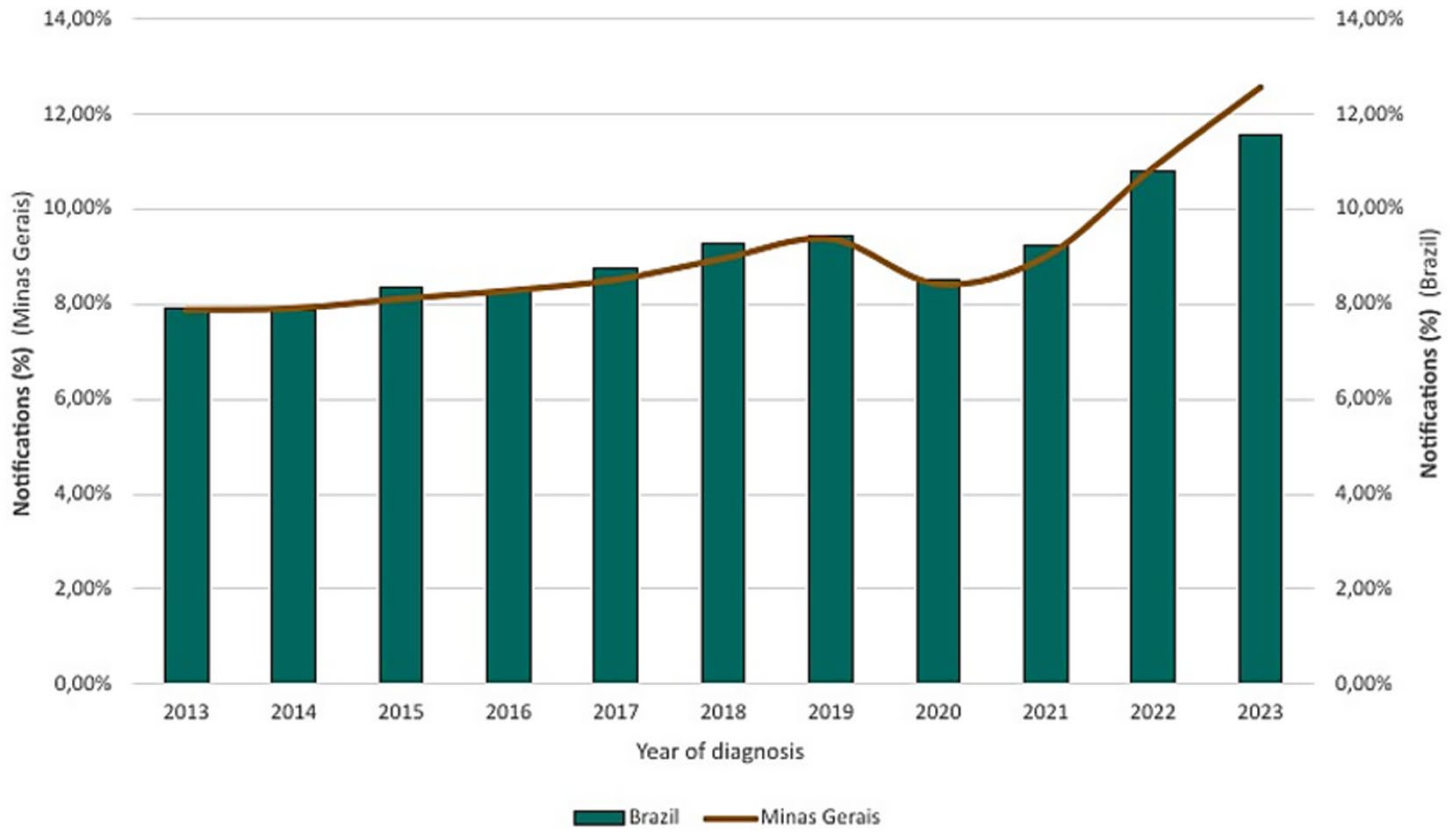
Confirmed tuberculosis cases in Brazil and Minas Gerais (2013–2023).

From 2013 to 2023, a total of 668,471 laboratory-confirmed cases of tuberculosis infection were reported in Brazil, with 31,341 cases in Minas Gerais. In Minas Gerais, 2023 presented the highest number of notifications with 3,932 (12.55%) cases, following a brief decline during 2020 and 2021 with 2,644 (8.44%) and 2,825 (9.01%) notifications, respectively. A progressive increase resumed in 2022 with 3,406 (10.87%) cases before peaking in 2023 (Figure 1).

**Figure 2:**
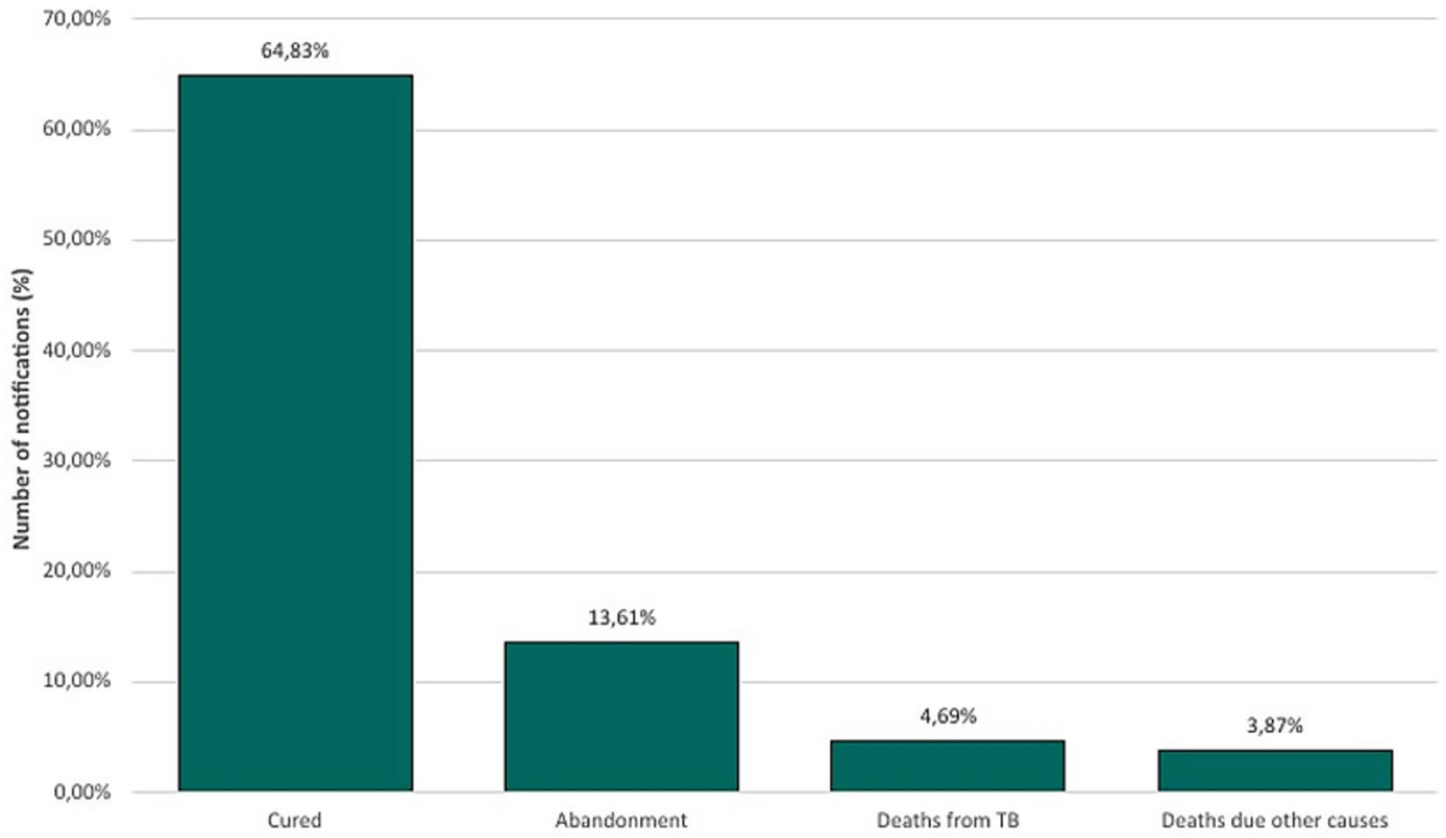
Closed cases of tuberculosis in Minas Gerais (2013–2023).

Among closed cases, Minas Gerais presented 20,319 (64.83%) cases successfully cured, with 1,469 (4.69%) deaths due to TB, while 1,214 (3.87%) deaths occurred due to other causes in people infected with TB. However, treatment abandonment rates were concerning, with 13.61% (4,266) of cases (Figure 2). The remaining closed cases are divided between ignored or blank notifications, transfer cases, drug-resistant tuberculosis, regimen changes, therapeutic failure, and primary abandonment.

**Figure 3:**
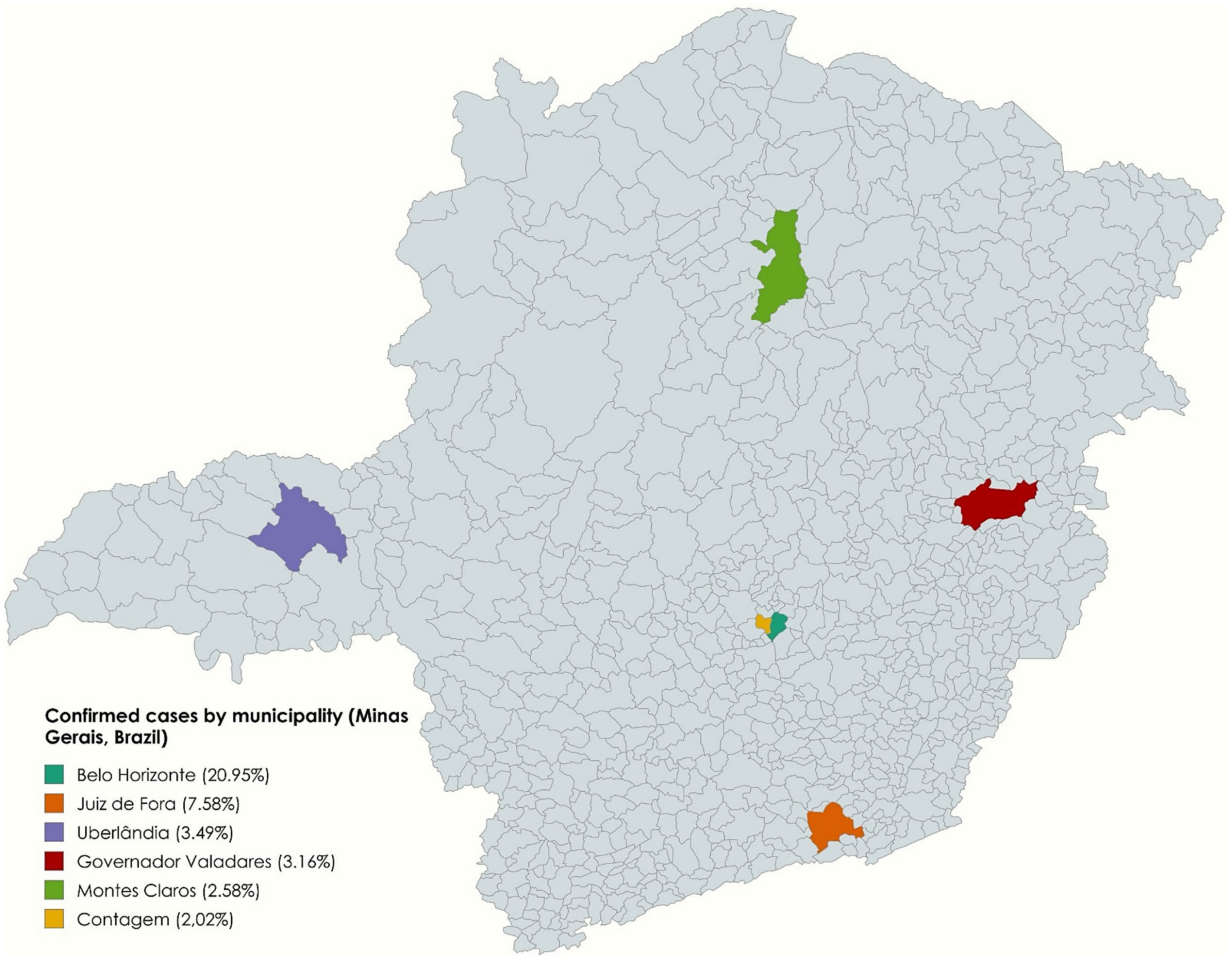
Geographic disparities in tuberculosis: the six municipalities with the highest number of confirmed cases, Minas Gerais, Brazil (2013–2023).

During the entire period analyzed in Minas Gerais, the municipality of Belo Horizonte presented, significantly, the highest number of notifications (6,567–20.95%), followed by the municipalities of: Juiz de Fora (2,377–7.58%), Uberlândia (1,094–3.49%), Governador Valadares (991–3.16%), Montes Claros (810–2.58%) and Contagem (633–2.02%) (Figure 3). Based on the 2022 Census published by the Brazilian Institute of Geography and Statistics (IBGE) (7), case rates per 1,000 inhabitants were calculated and detailed in Table 1.

**Table 1:** Reported cases, population and cases per 1,000 inhabitants in the municipalities with the highest number of notifications in Minas Gerais, Brazil.

Municipalities	Reported cases	População	Population cases per 1,000 inhabitants
Belo Horizonte	6,567	2,315,560	284
Juiz de Fora	2,377	540,756	44
Uberlândia	1,094	713,224	153
Governador Valadares	991	257,171	385
Montes Claros	810	414,240	195
Contagem	633	621,853	102

Source: Ministry of Health/SVSA—Notifiable Diseases Information System—Sinan Net; IBGE, 2022.

**Figure 4:**
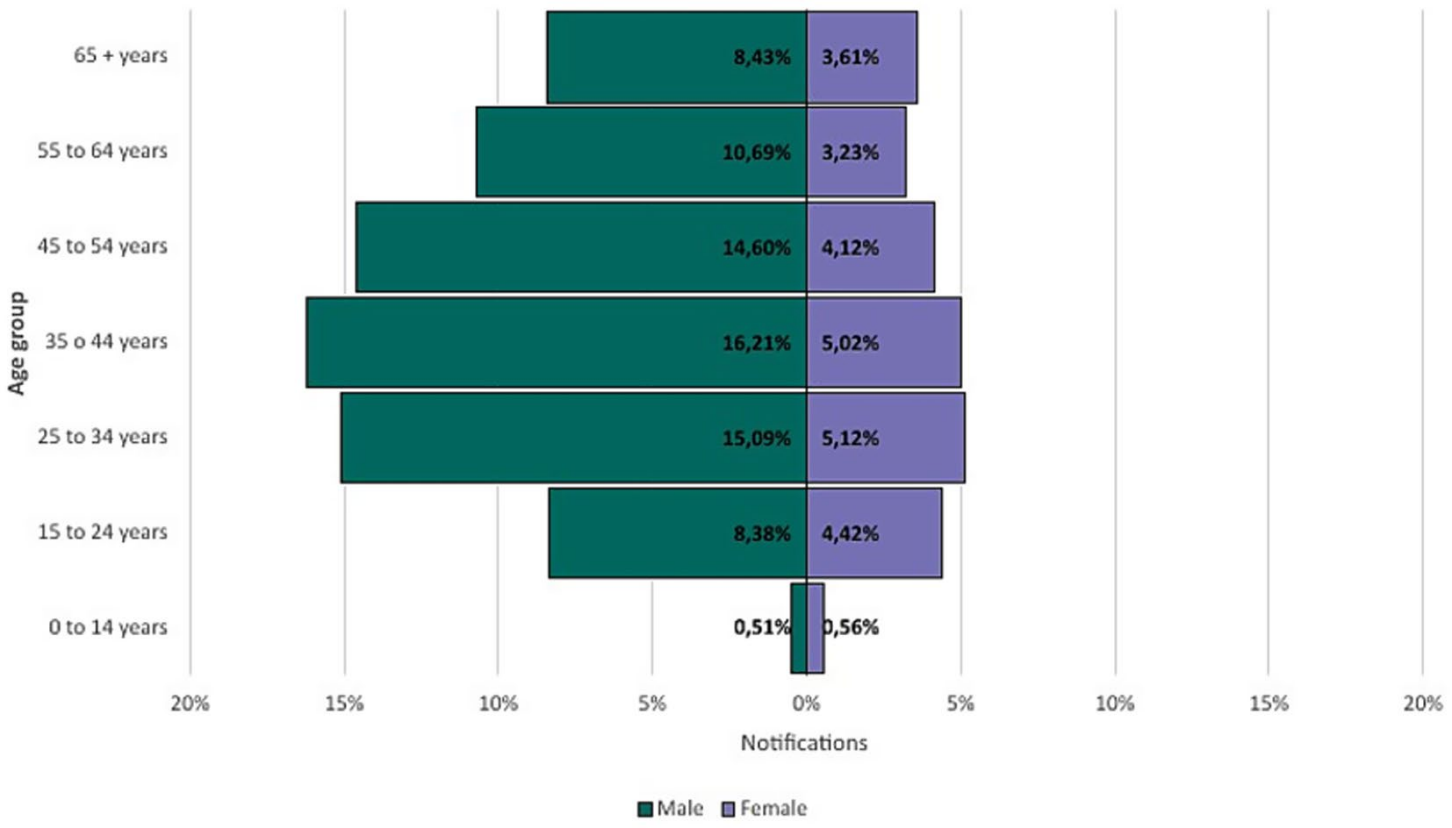
Age distribution of confirmed tuberculosis cases by sex in Minas Gerais (2013–2023).

When evaluating the notifications according to sex, in the state of Minas Gerais, male sex predominated, representing 73.91% (23,165) of the cases, while female sex represented 26.08% (8,174). Regarding the age group of the individuals, the 35–44 age group was most affected with 6,654 (21.23%) cases, followed by 25–34 years with 6,338 (20.22%) cases, 45–54 years with 5,868 (18.72%) cases, 55–64 years with 4,363 (13.92%) cases and 15–24 years with 4,011 (12.80%) cases. Individuals aged over 65 years accounted for 3,772 cases (12.03%), presenting considerably more cases than the ages under 14 years with 335 cases (1.07%) (Figure 4).

**Figure 5:**
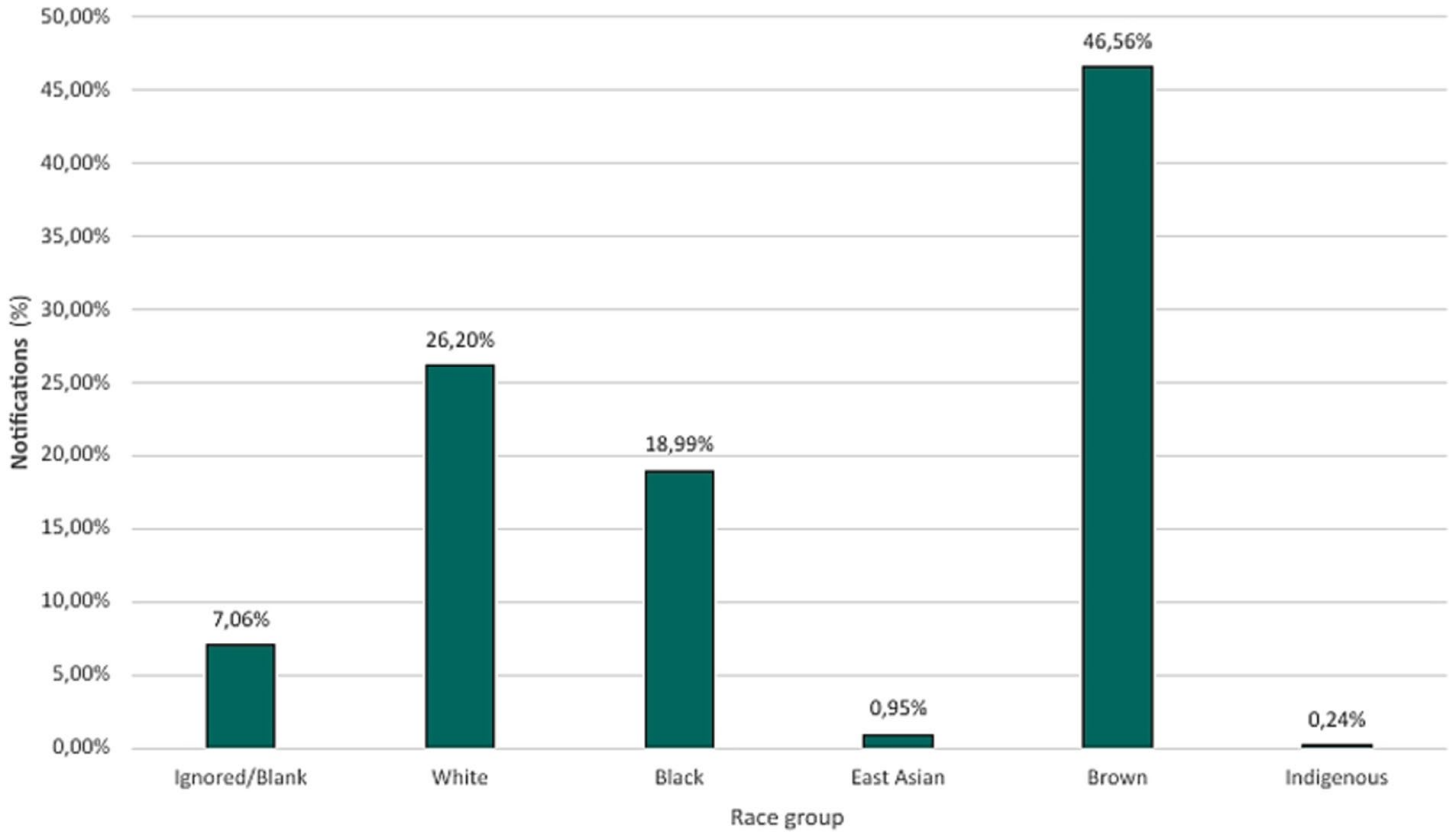
Percentage distribution of confirmed tuberculosis cases across racial groups (2013–2023).

Regarding races, data revealed that 14,592 (46.56%) of cases occurred among self-declared Brown-skinned (or Pardo in IBGE’s classification), followed by 8,212 (26.20%) cases of White individuals, 5,951 (18.99%) Black, 297 (0.95%) East Asian and 76 (0.24%) Indigenous, while 2,213 (7.06%) were reported blank or ignored. According to IBGE (7), the state of Minas Gerais is composed, approximately 46.8% of Brown-skinned, 41.1% White, 11.8% Black, 0.15% East Asian and 0.15% self-declared Indigenous (Figure 5).

**Figure 6:**
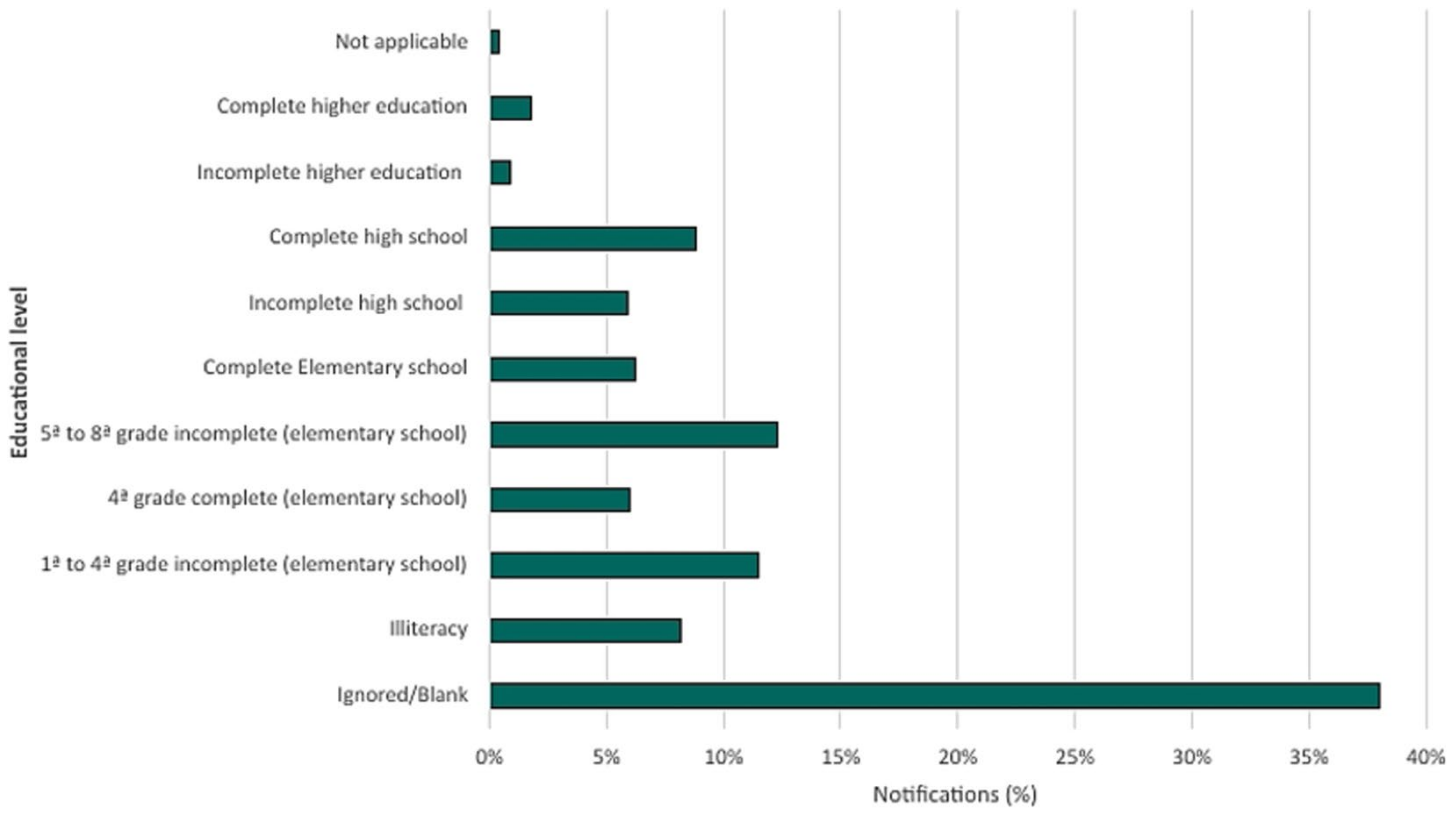
Percentage distribution of confirmed tuberculosis cases by educational level (2013–2023).

Educational attainment analysis showed that the highest number of notifications occurred in individuals with incomplete elementary education (5th–8th grade), with 3,865 (12.33%) cases, while 11,907 (37.99%) cases of individuals who lacked formal education or were illiterate. In contrast, the lowest number of notifications occurred in individuals with incomplete and complete higher education, totaling 831 cases (2.65%). Notifications listed as ignored or blank constitute 38.0% (11,911) of all cases (Figure 6).

**Figure 7:**
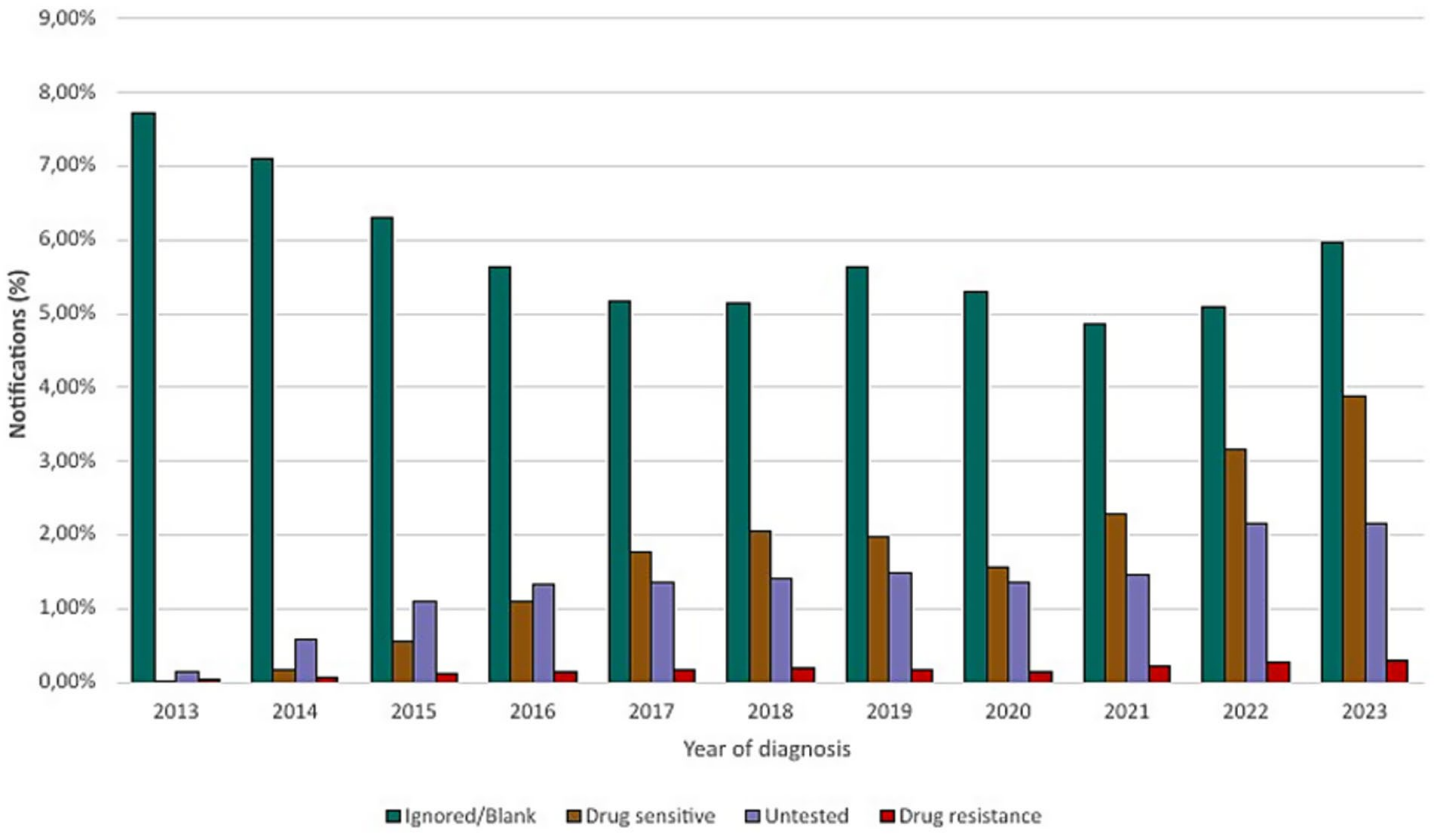
Drug susceptibility testing for tuberculosis (2013–2023).

**Figure 8:**
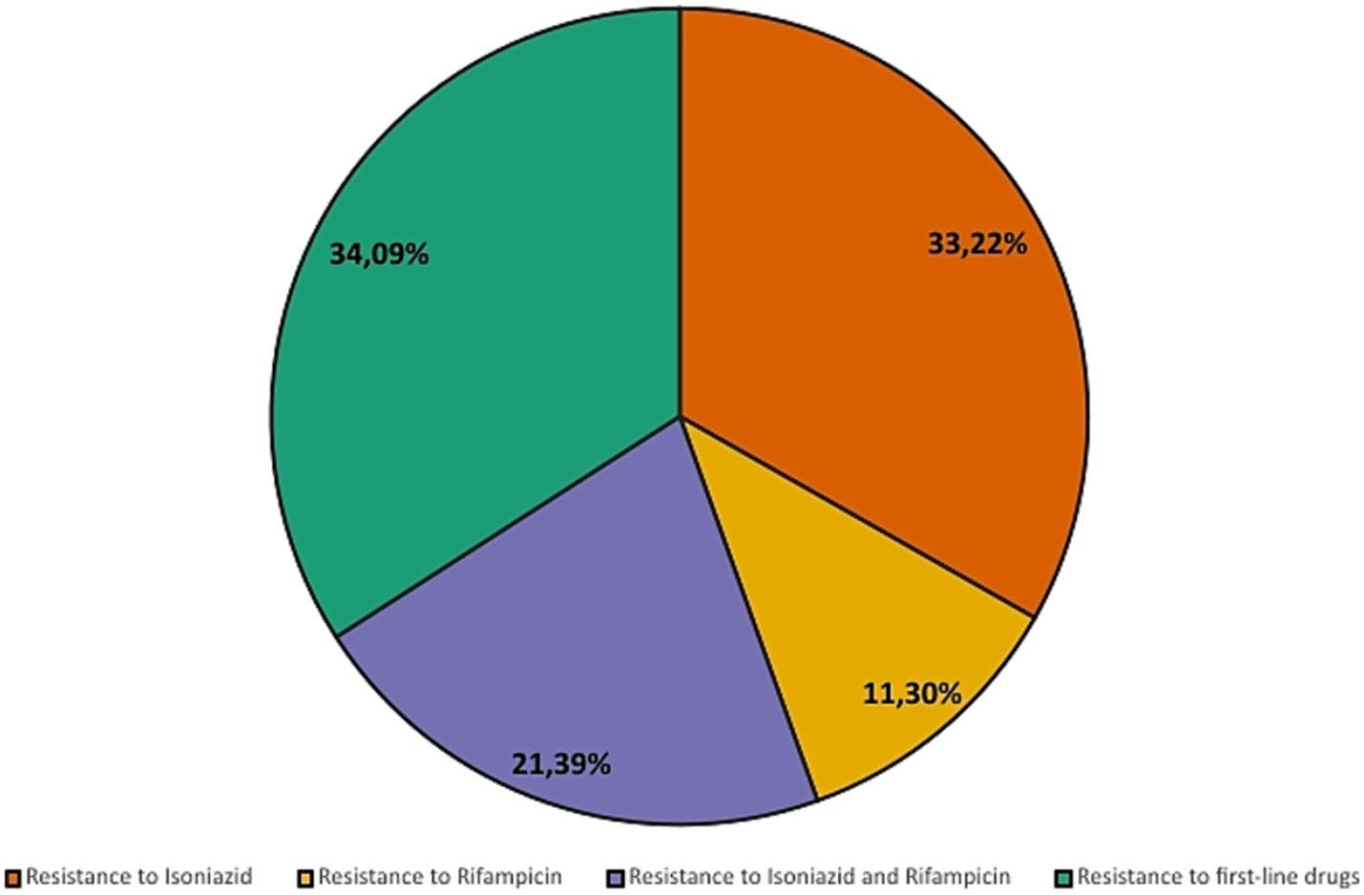
Percentage distribution of reported drug resistance profiles (2013–2023).

Notifications related to drug sensitivity testing demonstrated that, of the 31,341 cases in Minas Gerais, 18.49% (5,794) were sensitive to drug therapy, while 14.47% (4,536) of cases were untested and 1.83% (575) of cases presented drug resistance and the results for 1.29% (404) cases were pending. Among drug resistance, the most common was resistance to first-line drugs with 34.09% (196) followed by: resistance to isoniazid (33.22% or 191 cases); resistance to isoniazid and rifampicin simultaneously (21.39% or 123 cases); and resistance to rifampicin (11.30% or 65 cases), as shown in Figures 7, 8.

**Figure 9:**
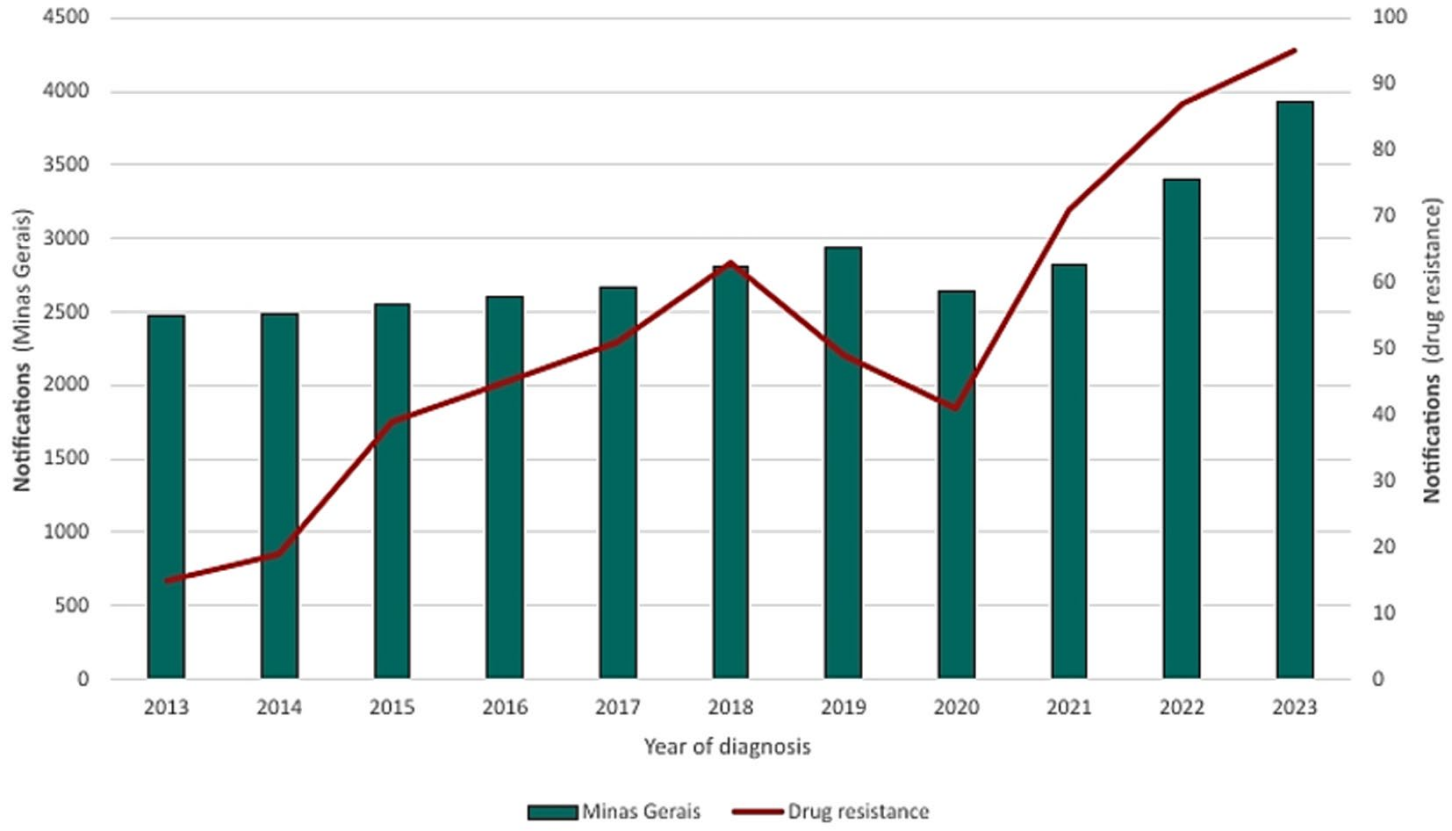
Confirmed cases and resistance patterns in Minas Gerais (2013–2023).

Between 2013 and 2023, the state of Minas Gerais reported an annual average of 2,500–4,500 new tuberculosis cases, with a peak recorded in 2023. During this period, only approximately 18–20% of reported cases underwent drug susceptibility testing, as reflected in the national rifampicin susceptibility testing coverage of around 18.5% in 2023. This limited testing coverage compromises the representativeness of resistance data. Nevertheless, an upward trend in rifampicin-resistant cases has been observed over the years. The confirmed cases of rifampicin resistance in Minas Gerais, during the study period, are presented in Figure 9.

**Figure 10:**
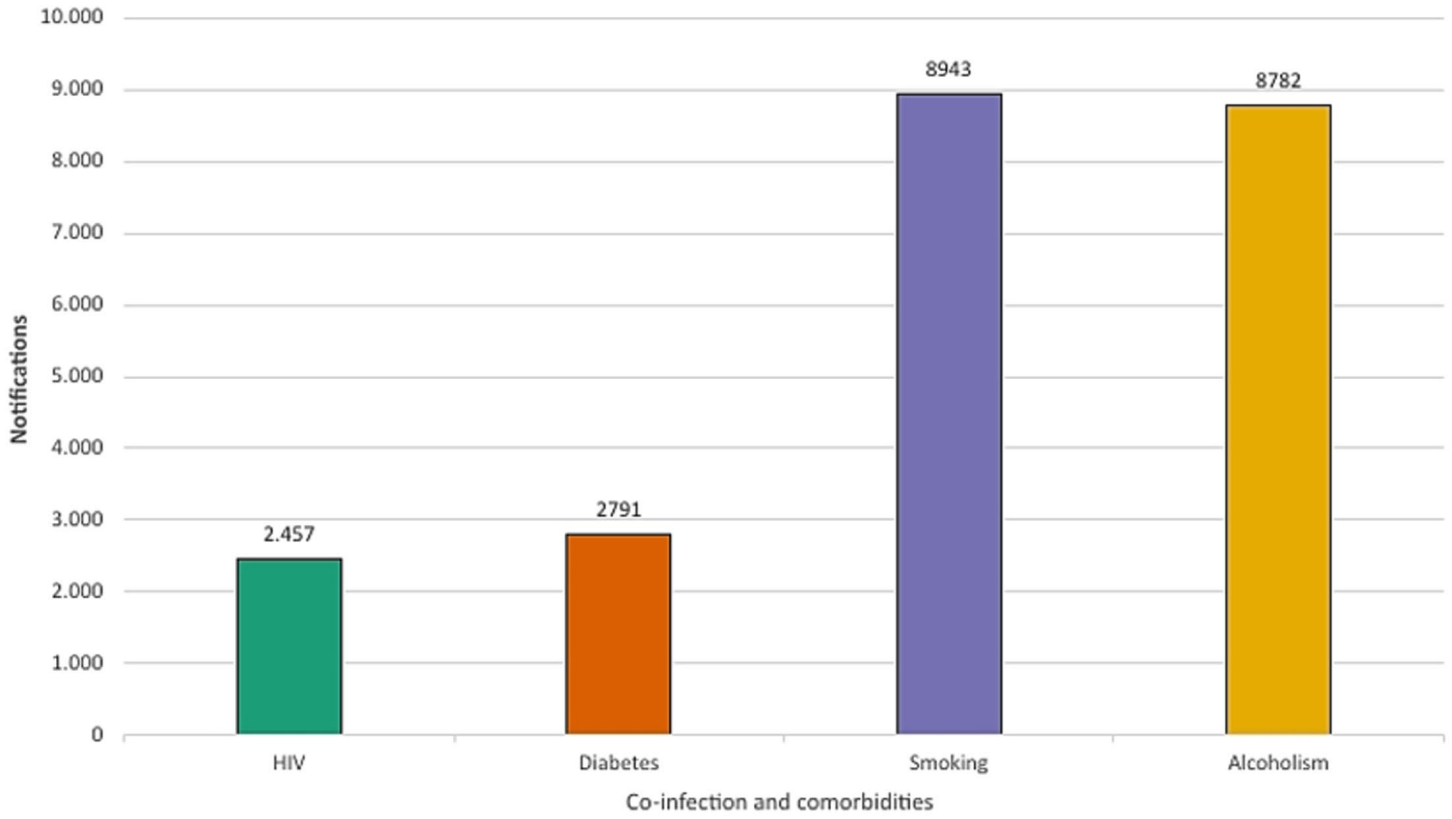
Prevalence of HIV co-infection and comorbidities among tuberculosis cases in Minas Gerais (2013–2023).

**Figure 11:**
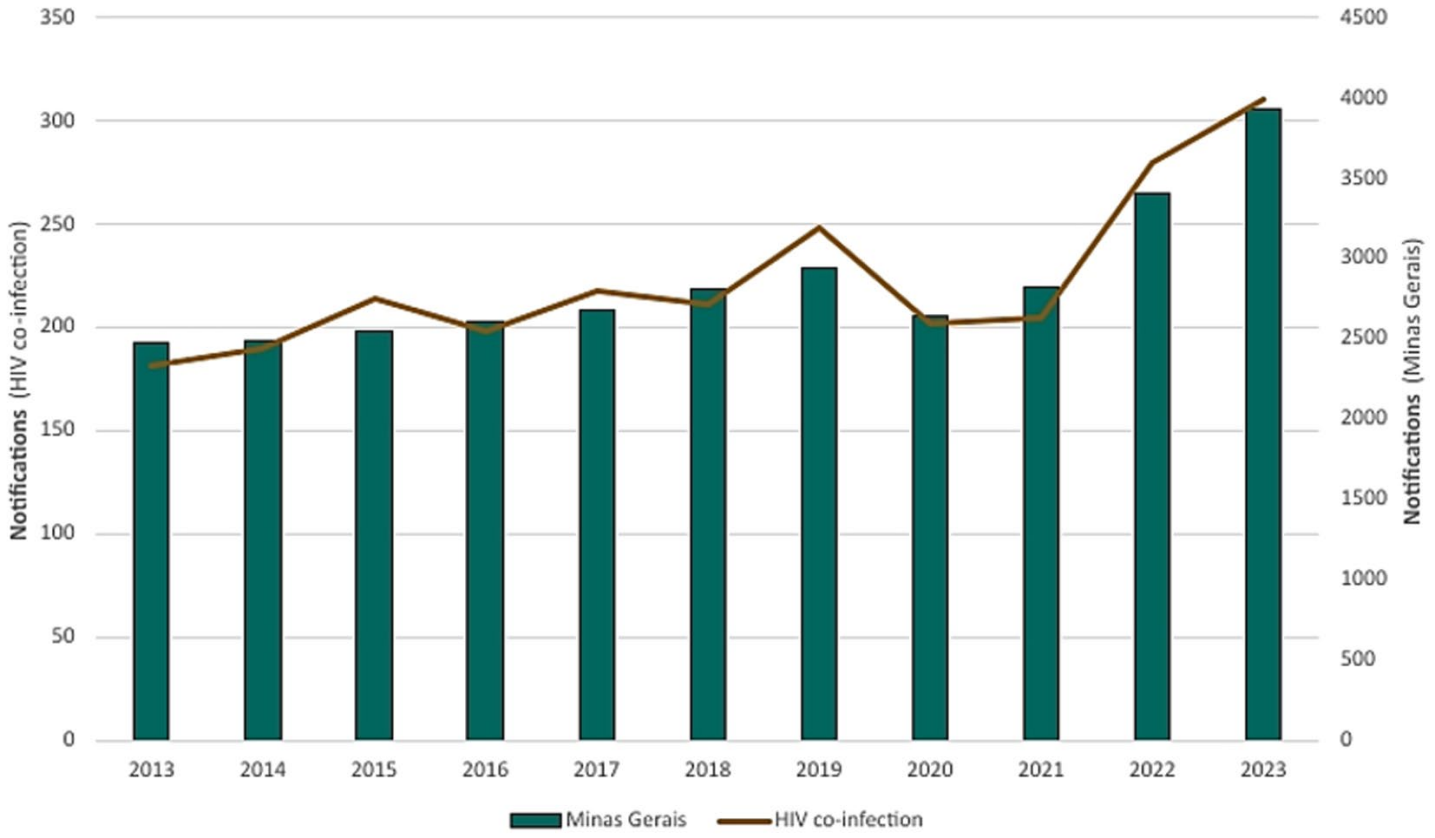
Burden of HIV co-infection among confirmed cases of tuberculosis in Minas Gerais (2013–2023).

HIV, frequently associated with tuberculosis, presented 2,457 (7.84%) cases of co-infection during the period analyzed, with incidence rates mirroring overall TB trends in Minas Gerais (Figures 10, 11).

**Figure 12:**
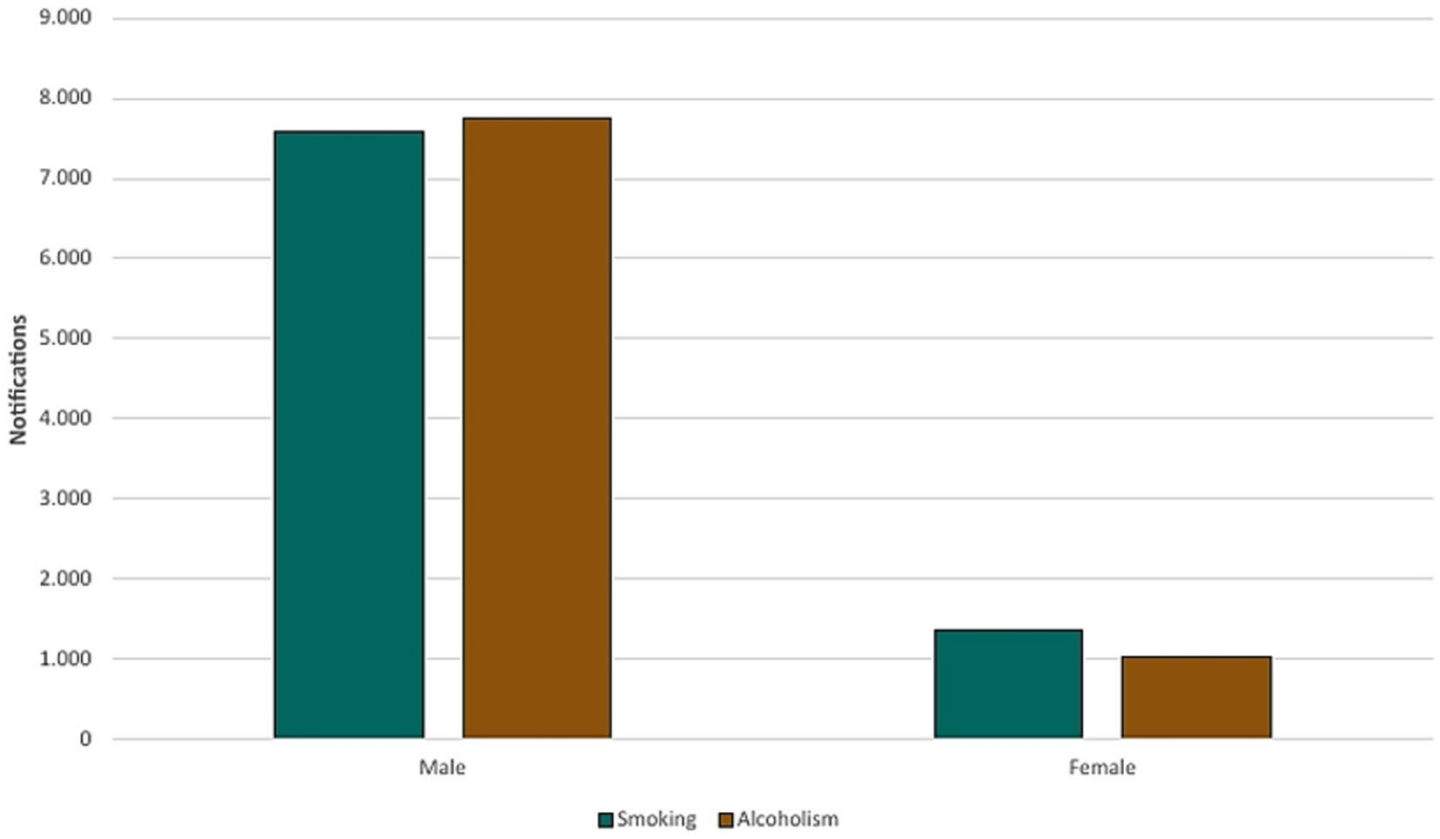
Relationship between smoking and alcohol use by sex (2013–2023).

Comorbidities and risk factors such as smoking, alcoholism and diabetes presented 8,943 (28.53%), 8,782 (28.02%), and 2,791 (8.90%) cases, respectively. Males comprised 84.66% (7,588) and 88.35% (7,759) of smoking and alcoholism-related cases, respectively, while female individuals comprised 15.34% (1,354) and 11.65% (1,023) (Figure 12).

## 4 Discussion

Tuberculosis is an infectious disease caused by *Mycobacterium tuberculosis*, which primarily affects the lungs but can compromise other organs and systems of the body. Transmitted via aerosols generated during speech, coughing, or sneezing by infected individuals, TB is considered one of the leading causes of death from infectious diseases worldwide. In 2021, approximately 10.6 million people developed tuberculosis globally, with 1.6 million associated deaths, highlighting its significance as a public health problem (8).

This study showed that, in Minas Gerais, there was a 4.64% increase in reported TB cases from 2013 to 2023. Contrasting with WHO’s “The end TB strategy” (2) target of a 50.0% reduction in incidence by 2025 compared to 2015, Minas Gerais showed a rise of 4.41% in incidence over the same period. Among the six municipalities with the highest number of notifications, only Governador Valadares is not among the six most populous in the state. According to 2022 IBGE (7) census data, Governador Valadares, with a population of 257,171 people, reported 3.85 cases per 1,000 inhabitants, the second highest incidence among analyzed municipalities. By comparison, Contagem, with a population of 621,863, more than double, reported 1.02 cases per 1,000 inhabitants, highlighting the disparities in case distribution. The same scenario extends to the municipality of Juiz de Fora, which presented the highest incidence among the municipalities analyzed, 4.4 cases per 1,000 inhabitants, with a smaller population than Belo Horizonte, Contagem, and Uberlândia.

It was possible to observe that most cases occurred among men (73.91%), predominantly those aged between 35 and 44 years, self-identified as Brown-skinned (46.56%), and with incomplete elementary education (5th–8th grade, 12.33%). Several factors contribute to this statistic, including the prevalence of males, the social and cultural context in which health is not a priority for men, which is one of them (9). Males accounted for 84.86% of TB cases involving smoking and 88.35% of alcohol use disorder, reinforcing behavioral risk factors.

These findings corroborate other studies that indicate the epidemiological profile of tuberculosis as men, of economically active age, and limited education, with a direct relationship with poverty and social exclusion, leading to abandonment of treatment (10). Epidemiological studies in Brazil indicate that men were twice as likely as women to develop TB (11). The disparity between sex is attributed to a combination of biological, behavioral and social factors. Men tend to have higher rates of exposure to risk factors, such as smoking and excessive alcohol consumption (1), which can compromise the immune system and increase vulnerability to infection by *Mycobacterium tuberculosis*. Furthermore, according to Horton et al. (12), men are only two-thirds of the proportion of women who seek public health services and taking 1.5 times longer to be diagnosed on average.

Diabetes proved to be an important risk factor, regardless of sex, present in 8.90% of all tuberculosis cases in Minas Gerais. The risk of developing active tuberculosis is three times higher among those with a hemoglobin A1c (HbA1c) level ≥7.0% than those with an HbA1c level <7.0%, and twice as high among patients with Diabetes Mellitus (DM) using ≥40 daily units of insulin (13, 14).

While the 35–44 age group was most affected, cases among those aged 25 and 54 years represented 60.17% of all cases. Tuberculosis can affect people of all ages, but its occurrence is higher among young and middle-aged adults, especially in the 20–40 age group, in low- and middle-income countries (8). The exposure of this group to work environments, social interactions, and community situations, in addition to the prevalence of comorbidities such as HIV (15), which are more frequent in economically active populations, creates a pattern. In high-income nations, on the other hand, cases concentrate among older adults due to population aging and latent TB reactivation in those individuals with a weakened immune system (16). These variations in the age distribution of cases reflect the influence of biological, social, and economic factors, highlighting the need for prevention and control strategies adjusted to the demographic characteristics of each region.

When assessing the race of individuals, a predominance of notifications was noted in those individuals who self-declared as Brown (46.56%); however, comparing with the data provided by the IBGE in Minas Gerais, which shows the predominance of Brown individuals in the state, a greater susceptibility related to race is excluded. Despite the proportionality, studies such as the one by Nery (17) indicate that poverty disproportionately affects Black and Brown communities, reaching 72.7% of Brazil’s poor. Poverty is a gateway to several other socio-environmental risks that increase the chances of developing the disease, such as inadequate sanitation, malnutrition, limited healthcare access, marginalization, the prison system, and low education (18, 19).

Therefore, tuberculosis presents significant differences in incidence between racial groups, influenced by socioeconomic factors, unequal access to health services, and social determinants. In many countries, populations belonging to historically marginalized racial groups are at higher risk of developing tuberculosis due to greater exposure to poor housing conditions, poverty, and malnutrition, as well as barriers to access to diagnosis and treatment (2, 15).

In this study, it was observed that 37.99% of individuals with TB infection did not have completed elementary school, a concerning trait, especially when compared with those with higher education, with 2.65% of cases, the lowest-incidence group. This data proves the relation between educational level and chances of developing the disease, despite the majority of notifications being presented as ignored or blank (38.0%). Tuberculosis is more prevalent in populations with low levels of education, reflecting the interaction between social determinants and conditions of vulnerability. Individuals with lower levels of education often face barriers in accessing information about prevention, early diagnosis, and treatment adherence, in addition to being more exposed to risk factors such as poverty, exacerbating this disparity (15).

Treatment abandonment and inappropriate use of medications are an even greater problem. In Brazil, 96.0% of drug resistance cases are acquired (20), that is, a strain initially sensitive to treatment develops resistance during therapy. In Minas Gerais, although the results of this study indicate only 1.83% of cases as presenting some type of resistance, the rise in cases between 2013 and 2023 is cause for concern. In 2013, 2.475 cases of tuberculosis were reported, with 0.61% showing resistance. By 2023, this number rose to 2.42% (of 3,932 cases), an increase of 1.81%.

*Mycobacterium tuberculosis* resistant to rifampicin (RR-TB) represents a serious challenge in global tuberculosis control, as it is associated with high rates of morbidity, mortality, and costs for healthcare systems. Rifampicin resistance is often used as a marker for multidrug resistance, including isoniazid, characterizing multidrug-resistant tuberculosis (MDR-TB). This resistance arises primarily from mutations in the rpoB gene, which encodes the beta subunit of bacterial RNA polymerase, a target of rifampicin (8, 21). Between 2013 and 2023, signs of increasing resistance to rifampicin were observed in tuberculosis cases reported in Minas Gerais, although only a limited percentage of samples were subjected to susceptibility testing (around 18–20% of cases). The results showed a decrease in resistance in 2020, but this result should be analysed with caution due to low testing coverage and the possibility of selection bias during the COVID-19 pandemic.

The indiscriminate use of antibiotics in hospitalized patients with COVID-19 has been reported by the World Health Organization and identified as a potential factor contributing to the rise in bacterial resistance. In the state of Minas Gerais, Brazil, cases of rifampicin-resistant tuberculosis in 2022 were 43.68% higher than in 2019 and 52.87% higher than in 2020. The reduction observed in 2020 may be attributed to underreporting during the peak of the pandemic. Nonetheless, the post-pandemic increase remains substantial. It is important to note, however, that only a limited proportion of tuberculosis cases underwent drug susceptibility testing, which constrains the generalizability of these findings. Therefore, the association between increased resistance and factors such as excessive antibiotic use or treatment interruptions during the pandemic should be interpreted with caution and warrants further investigation. These observations underscore the relevance of tuberculosis on the WHO list of priority pathogens (22).

The COVID-19 pandemic severely disrupted tuberculosis surveillance systems worldwide. In the Global Tuberculosis Report 2023 (1), the WHO highlights an upward trend in the number of cases worldwide between 2017 and 2019, and an 18% decline between 2019 and 2020. In Minas Gerais, this trend mirrored the global pattern, with cases falling by 10%, from 2,940 to 2,644 cases. In 2022, both globally and in Minas Gerais, TB notifications rebounded sharply, beyond pre-COVID years. Worldwide, there was an increase of 16% above the number of cases in 2021 and 28% compared to 2020, while in Minas Gerais, cases in 2022 increased by 17.06% compared to 2021 and 22.37% compared to 2020.

Tuberculosis control programs have been significantly impacted by the COVID-19 pandemic, exacerbating existing challenges in combating this disease. The overload of healthcare systems due to the increase in COVID-19 cases led to delayed TB diagnoses, interrupted treatments, and reduced active case finding (1). In addition, co-infection with SARS-CoV-2 and *Mycobacterium tuberculosis* can exacerbate the clinical presentation of both cases, since COVID-19 can weaken the immune system, making tuberculosis patients more susceptible to severe forms of the disease (23).

HIV co-infection rates were another important parameter observed. The risk of a person developing active TB is 26 times higher than those without HIV (2). This association is a major public health problem and one of the main causes of TB mortality. The results showed that TB/HIV co-infection accounted for 7.84% of all cases between 2013 and 2023. In 2022, the co-infection accounted for 8.22% of all tuberculosis cases in Minas Gerais, excluding a direct impact of the pandemic, since a similar percentage was recorded in 2019 (8.43%). Worldwide, this rate was 6.3% in 2022 (1).

Despite the significant findings, the data gap during the pandemic, mainly attributed to the overload of healthcare systems and the interruption of some essential services, may have masked the magnitude of the problem. In the studied state, the apparent decline in the notification of new cases, even with continued transmission indicators, suggests an underreporting increase. The reallocation of professionals, suspension of screening campaigns, and the population’s fear of seeking appropriate care due to the risk of contamination by the coronavirus disease explain this scenario (24, 25). The lack of diagnosis not only hindered early identification but may also have facilitated its transmission due to the lack of adequate treatment, contributing to the increase in notifications in the post-pandemic period.

Tuberculosis is a serious public health problem in Brazil, with a significant burden of morbidity and mortality, especially in vulnerable populations. In 2021, the country recorded an incidence rate of 32.4 cases per 100,000 inhabitants and a mortality rate of 2.6 deaths per 100,000 inhabitants, reflecting challenges in early diagnosis and treatment adherence (26). The disease is largely influenced by social determinants, such as poverty, malnutrition, urban agglomerations, and HIV co-infection, which increase susceptibility to *Mycobacterium tuberculosis* infection. Strategies such as strengthening the Primary Health Care (the first contact between the patients and the public healthcare system in Brazil) and the use of molecular technologies for diagnosis have been implemented to achieve the goals of eliminating tuberculosis by 2035, as recommended by the World Health Organization (WHO) (2).

## 5 Study limitations

This study presents some limitations inherent to the use of secondary data from DATASUS/SINAN. First, tuberculosis case definitions were not limited to bacteriologically confirmed cases but also included those reported based on clinical, radiological, or histopathological criteria, according to the information available in the database. This heterogeneity may affect the accuracy of the epidemiological analysis.

Another important aspect to consider is the variable completeness of the data, particularly in fields such as education and race/skin color, which showed high proportions of missing or “ignored” entries. These gaps limit the interpretation of potential social inequalities in the case profile.

Furthermore, no statistical imputation was performed for missing data; instead, a descriptive analysis was conducted using the available information. This choice may affect the representativeness of certain sociodemographic variables and should be considered when interpreting the results.

Finally, it is important to note that because the data were aggregated rather than individualized, it was not possible to control confounding variables or conduct robust causal analyses on the impact of the COVID-19 pandemic. This limitation justifies the predominantly descriptive approach adopted in this study. We recommend that future research employing more complete, individual-level data apply more robust analytical methods to better elucidate the social determinants of tuberculosis.

## 6 Conclusion

The data collected in this study enabled the identification and confirmation of the epidemiological profile of tuberculosis, which predominantly affects male individuals of Brown skin (Pardo), aged 25–54 years, with low educational attainment. These epidemiological findings align with the already established epidemiological pattern of the disease, highlighting these socioeconomic conditions as an important risk factor. Thus, it is possible to relate the prevalence of the disease directly to education level and indirectly associated with race within the social context of Minas Gerais state.

Reported cases of HIV/TB co-infection were not directly impacted by the COVID-19 pandemic, maintaining consistent incidence rates in both pre- and post-pandemic years. However, the COVID-19 pandemic had a significant impact on epidemiological surveillance systems, exacerbating underreporting and the rising number of cases involving drug resistance.

The epidemiological evidence from this study reaffirms tuberculosis as a critical public health problem, disproportionately affecting those in social vulnerability, while the increase in incidence over the study period contradicts the WHO’s expectations for the end of the global tuberculosis epidemic.

This study highlights the importance of public health data systems for epidemiological surveillance, enabling the identification and the impact of multiple risk factors and external determinants for decision-making purposes and the development of new public policies for disease control.

Given this scenario, it is essential to strengthen epidemiological surveillance efforts, expand access to early diagnosis and molecular testing, and implement intervention strategies tailored to local realities and the most vulnerable groups. Integration between primary care services, tuberculosis control programs, and social initiatives must be prioritized to contain the spread of the disease and mitigate its impacts in the coming years.

## Data Availability

The raw data supporting the conclusions of this article will be made available by the authors, without undue reservation.

## Footnotes

^1^ http://datasus.saude.gov.br
